# Topological and Functional Characterization of an Insect Gustatory Receptor

**DOI:** 10.1371/journal.pone.0024111

**Published:** 2011-08-29

**Authors:** Hui-Jie Zhang, Alisha R. Anderson, Stephen C. Trowell, A-Rong Luo, Zhong-Huai Xiang, Qing-You Xia

**Affiliations:** 1 The Key Sericultural Laboratory of Agricultural Ministry, Southwest University, Chongqing, China; 2 CSIRO Food Futures Flagship, Canberra, Australian Capital Territory, Australia; 3 CSIRO Ecosystem Sciences, Canberra, Australian Capital Territory, Australia; 4 Institute of Agronomy and Life Science, Chongqing University, Chongqing, China; AgroParisTech, France

## Abstract

Insect gustatory receptors are predicted to have a seven-transmembrane structure and are distantly related to insect olfactory receptors, which have an inverted topology compared with G-protein coupled receptors, including mammalian olfactory receptors. In contrast, the topology of insect gustatory receptors remains unknown. Except for a few examples from *Drosophila*, the specificity of individual insect gustatory receptors is also unknown. In this study, the total number of identified gustatory receptors in *Bombyx mori* was expanded from 65 to 69. BmGr8, a silkmoth gustatory receptor from the sugar receptor subfamily, was expressed in insect cells. Membrane topology studies on BmGr8 indicate that, like insect olfactory receptors, it has an inverted topology relative to G protein-coupled receptors. An orphan GR from the bitter receptor family, BmGr53, yielded similar results. We infer, from the finding that two distantly related BmGrs have an intracellular N-terminus and an odd number of transmembrane spans, that this is likely to be a general topology for all insect gustatory receptors. We also show that BmGr8 functions independently in *Sf9* cells and responds in a concentration-dependent manner to the polyalcohols *myo*-inositol and epi-inositol but not to a range of mono- and di-saccharides. BmGr8 is the first chemoreceptor shown to respond specifically to inositol, an important or essential nutrient for some Lepidoptera. The selectivity of BmGr8 responses is consistent with the known responses of one of the gustatory receptor neurons in the lateral styloconic sensilla of *B. mori*, which responds to *myo*-inositol and *epi*-inositol but not to *allo*-inositol.

## Introduction

Despite detailed morphological and physiological knowledge of gustation in insects [1], [2], [3], [4], [5], this understanding does not extend to the molecular level. The recent identification of gustatory receptor (GRs) sequences from whole genome sequencing has enabled investigations of molecular gustation in insects. Insect GRs and olfactory receptors (ORs) were originally classified as a large subfamily of G protein-coupled receptors [6], [7], [8], [9]. Subsequent studies have shown that insect ORs have an inverted transmembrane structure and can signal independently of heterotrimeric G proteins *in vitro* [10], [11], [12], [13], [14], although olfactory signal transduction has been shown also to involve heterotrimeric G-proteins [15]. The transmembrane topology and signalling mechanisms of insect GRs remain unknown although there is evidence of both a G-protein independent pathway and a G-protein dependent pathway [16], [17], [18], [19].

Insect gustatory receptors have been classified into “sugar” and “bitter” clades based on their homologies with identified receptors from *Drosophila*. *DmGr5a* was reported as a trehalose specific receptor [20], [21], [22] and members of the *DmGr64* cluster (*DmGr64a-f*), which are orthologs of *DmGr5a,* have been reported to be important in the detection of sucrose, maltose and glucose [22], [23]. *DmGr64f* is required for detection of a range of sugars and it is thought to act as a co-receptor for other sugar receptors [24]. A number of *Drosophila* GRs have also been implicated in the responses to caffeine and other bitter plant secondary metabolites [25], [26], [27], [28]. Based on homologies to these receptors, the genome of *Drosophila melanogaster* [29] contains eight putative sugar receptors. Against this background the first fully sequenced lepidopteran genome of *B. mori*, encodes five putative sugar receptors [30], [31]. The *Bombyx* genome also contains a large number of putative bitter receptors. Despite almost 60 putative sugar GRs being identified from insects, ligands have only been assigned to a few of the *Drosophila* sequences [32].

In this study, we identified the topological structure of two insect GRs. Analysis of *BmGr8* suggests that it has an uneven number of transmembrane segments and that its N-terminus is intracellular and its C-terminus is extracellular. We observed a similar topology for the bitter receptor candidate, *BmGr53*, suggesting that insect GRs have the same transmembrane topology as insect ORs. In addition to the topological structure analysis we show the putative sugar receptor, *BmGr8,* responds selectively to *myo*-inositol at physiologically relevant concentrations.

## Materials and Methods

### Bioinformatics

New assemblies of the *Bombyx mori* genome sequence were searched with known annotated silkworm GRs [31] and other insect GRs as queries using TBLASTN [33]. The genomic scaffold sequences containing candidate genes were predicted using FGENESH+ (http://www.softberry.com/berry.phtml), BGF (http://bgf.genomics.org.cn/) and SplicePredictor (http://deepc2.psi.iastate.edu/cgi-bin/sp.cgi). Multiple transmembrane domains of receptors were predicted by TMPred (http://www.ch.embnet.org/software/TMPRED_form.html), TMHMM (http://www.cbs.dtu.dk/services/TMHMM-2.0/) and HMMTOP (http://www.enzim.hu/hmmtop/html/submit.html/) (Table S1).

Phylogenetic analysis was conducted with the 69 BmGrs and putative gustatory receptors from *Heliothis virescens* [34], putative sugar receptors from *Drosophila melanogaster* [29], *Anopheles gambiae* [35], *Apis mellifera* [36], *Aedes aegypti* [37], [38], *Tribolium castaneum* [39], [40], *Nasonia vitripennis* [41], *Acyrthosiphon pisum* [42] and *Culex quinquefasciatus* [42], [43]. Protein sequences of GRs were downloaded from NCBI. All sequences used for phylogenetic analysis may also be downloaded from http://silkworm.swu.edu.cn/silkdb/doc/download.html. Amino acid sequences were aligned with Clustal X using default parameters (alignment of insect sugar receptors is shown in Figure S1) and subjected to phylogenetic analysis in order to demonstrate their evolutionary relationships. With RAxML [44], the maximum likelihood (ML) tree was constructed with Blosum62 implemented as the amino-acid model. A rapid bootstrapping analysis was conducted using 1000 replicates, with other parameters set to their default values. The Bayesian tree was generated with the software MrBayes v.3.1.2 [45], in which the *Blosum62* [46] model was used. Two independent Markov Chain Monte Carlo (MCMC) analyses of 10 million iterations were performed, each with 4 chains (three hot and one cold), sampling one tree per 1000 iterations with the burnin percentage as 0.25. Posterior probabilities were used to estimate the reliability of the Bayesian tree topology.

### Insects and receptor gene cloning and sequencing

*Bombyx mori* larvae from the inbred domesticated *Dazao* strain [47] were reared on mulberry leaves at 25°C by the Key Sericultural Laboratory of the Agricultural Ministry, Southwest University [47]. GRs were amplified from *B. mori* maxillary galeal cDNA using gene specific primers. The 5′–3′ nucleotide sequences of all primers used in this study are listed in Figure S2. RNA was prepared using Trizol (Invitrogen, USA) according to the protocol provided by the manufacturer and was treated using Dnase I. First strand cDNA was synthesized using MMLV Reverse Transcriptase following the recommended protocols (Promega, USA). Remaining RNA was subsequently digested using RNase H (MBI Fermentas). Amplified genes were cloned into pGEM-T vector (Promega, USA) and subcloned into PIB/V5-His vector (invitrogen, USA) using Kpn I/Sac II. Plasmid DNAs were sequenced by Micromon (Monash University).

To detect the expression of *BmGr8* in *Sf9* cells, and to assess the topology of GRs, two copies of the MYC-epitope tag, comprising the amino acids EQKLISEEDL, were incorporated in frame either upstream or downstream of both *BmGr8* and *BmGr53*. Appropriate restriction sites (Sac II/Kpn I for *BmGr8*; Xba I/BamH I for *BmGr53*) were introduced to allow cloning into the PIB/V5-His vector (Figure S2).

### Cell culture and transient transfection

*Spodoptera frugiperda Sf9* cells (Invitrogen) were maintained as a suspension culture in Sf-900 II medium (Invitrogen) according to the manufacturer's instructions. Cells were plated into 12-well plates and left to settle for half an hour before being transiently transfected with 500ng of plasmid construct and 3 µL of Escort IV transfection reagent (Sigma, USA) in 100 µL of medium per well. After incubation for 7 h, the cells were washed twice with fresh medium and incubated in 1 mL of fresh medium until needed.

*Drosophila* Schneider S2 cells (Invitrogen, USA) were maintained as a suspension culture in *Drosophila* Schneider's medium (Invitrogen, USA) supplemented with 10% fetal bovine serum (Invitrogen, USA). Cells were subcultured onto poly-L-Lysine coated coverslips in 6-well plates and transfected using Fugene HD transfection reagent (Promega, USA) according to the product manual. After transfection, the coverslips were washed and incubated with 2 mL fresh medium at 28°C for 48 h before detection of protein expression.

### Calcium Imaging

*Sf9* cells were transfected as described above. Calcium imaging was modified from the method performed previously [13], [48] to avoid high levels of D-glucose in the assay buffer. The cells were transfected with PIB/V5-His vector (control), BmGr8 or MYC/BmGr8. After forty-eight hours post transfection, the medium was removed and the cells were washed with assay buffer. Cells were loaded with calcium indicator mix containing 2 µM Fluo4 (Invitrogen, USA), 1.5 µL Pluronic acid in 250 µL assay buffer and incubated at 28°C for 20 minutes. Assay buffer was modified from Hanks' Balanced Salt Solution as 5 mM KCl, 130 mM NaCl, 0.5 mM MgSO_4_, 2 mM CaCl_2_, 1 mM NaHCO_3_, 10 mM D-glucose, 2 mM probenecid, 10 mM HEPES (pH 7.4) and sterilized through a 0.22 µm filter. The cells were washed twice with saline to remove excess dye and were covered with 400 µL of fresh buffer. Each plate was incubated in the dark for a further 20 min prior to calcium imaging. The cells were tested with the eleven tastants listed in the next sub section. Fluorescence imaging was conducted on a Leica inverted microscope (Leica, Germany) and a Leitz digital still camera. Images were recorded every 10 s for 50 s following the addition of assay buffer (negative control), the test ligand and ionomycin (to determine maximal fluorescence). Fluorescence intensity was recorded for identified cells in each image using the Metafluor® imaging system. ΔF was calculated as the ratio of change in fluorescence from basal levels (saline) upon the addition of ligand relative to change in fluorescence from basal levels following the addition of ionomycin. Cells responding to saline alone were omitted from subsequent analysis as well as a small population of cells that were hyper-responsive to test substances. The responses of cells transfected by empty vector were subtracted from those transfected by test vectors. All points on log concentration-response curves are means of ΔF calculated using at least 30 cells. Data were analyzed and graphed with GraphPad Prism 5 using the two-tailed Student's t-test for comparisons between two groups. The data was arcsine transformed before the t-test was applied.

### List of tastants

The tastants tested were D-fructose, D-galactose, D-glucose, sucrose, D-maltose, D-trehalose, *myo*-inositol, *allo*-inositol, *scyllo*-inositol and methyl-α-D-glucopyranoside, which were all purchased from Sigma-Aldrich. *Epi*-inositol was purchased from TCI (Tokyo, Japan). The typical maximal and final concentrations of tastants in each well are as follows: D-fructose (50 mM), D-galactose (50 mM), D-glucose (50 mM), sucrose (50 mM), D-maltose (50 mM), D-trehalose (50 mM), methyl-α-D-glucopyranoside (50 mM), *myo*-inositol (75 mM), *allo*-inositol (50 mM), *scyllo*-inositol (50 mM) and *epi*-inositol (75 mM).

### Immunocytochemistry

Immunocytochemistry under permeabilised and non-permeabilised conditions was modified from Smart et al. (2008) [13]. All steps were performed at 4°C unless otherwise stated. 48 h after transfection, coverslips with S2 cells were transferred to a clean 6-well plate and washed three times for 5 minutes each with 1x PBS (pH 7.2). Cells were fixed in 2% formaldehyde solution in 1x PBS for 15 minutes and rinsed in 50 mM NH_4_Cl in 1x PBS. The cells were blocked in blocking buffer (1x PBS with 5% goat serum), either with or without 0.1% (w/v) saponin permeabilisation, for 15 min. Cells were incubated in a 1∶200 dilution of mouse anti-myc antibody (R950-25, Invitrogen) in blocking buffer for 2 h, washed with 1x PBS, incubated in 1∶200 dilution of Alexa Fluor® 488-tagged goat anti-mouse IgG (A-11001 Invitrogen) in blocking buffer for 1 h, washed with PBS and incubated in 1x PBS containing 2 µg/mL of the nuclear counterstain 4,6-diamidino-2-phenylindole (DAPI), for 5 minutes. The cells were briefly rinsed in 1x PBS before observing fluorescence using a Leica SP2 confocal laser scanning microscope (Leica, Germany).

### β-galactosidase fusion expression for topology study

β-galactosidase fusion is often used to study the location and topology of transmembrane proteins [10], [49], [50] as β-galactosidase is only functional when expressed inside a cell. To generate the constructs for expression of β-galactosidase fusions, *LacZ* was amplified from the pSV-β-Galactosidase control vector (Invitrogen, USA) using a forward primer with a *TrpS* linker (Figure S2). *TrpS:LacZ* was digested with Xho I and Sac II and directionally cloned into the PIB/V5-His vector. Gene fragments of *BmGr8* (AA1-28, AA1-62, AA1-75), *BmGr53* (AA1-64, AA1-96), *rhodopsin* (*RH1*) (AA1-49, AA1-42) and corresponding controls with each fragment fused to a downstream synthetic transmembrane domain were synthesised by GenScript (USA). A construct containing the coding sequence of LacZ, with the start codon deleted, was used as a negative control for endogenous galactosidase activity. The gene fragments were subcloned upstream of *TrpS:LacZ* in PIB/V5-His using Kpn I/Xho I. The sequences of TrpS linker and synthetic transmembrane domain were based on the pPD34.110 construct [50]. Plasmid DNAs were transfected into *S. frugiperda Sf9* cells using Escort IV transfection reagent (Sigma, USA) and also into *Drosophila* Schneider *2* (S2) cells (Invitrogen, USA) using Fugene HD transfection reagent (Promega, USA). Before assessing β-galactosidase activity, cells were rinsed in 1x PBS three times, fixed in 2% formaldehyde/0.2% glutaraldehyde in 1× PBS (pH 7.2) for 15 min, rinsed three times in 1 × PBS and incubated for three hours in X-gal staining solution at 37°C. Subsequently, X-gal solution was removed, cells were rinsed in 1 × PBS and observed with a light microscope. Cellc [51] and ImageJ (http://rsb.info.nih.gov/ij/) were employed to estimate the number of stained cells as a percentage of total cells. At least 2 000 cells were counted from each picture.

## Results

### Phylogenetic analysis of *B. mori* gustatory receptors

In order to select the receptors to study we performed a phylogenetic analysis of the *B. mori* GR family. By searching the 9× *B. mori* genome database [31], we identified four partial GR genes in addition to the 65 previously reported from this species [30], [31], The four additional receptors are named BmGr66-69. The sequences of all 69 BmGrs together with those of the putative sugar receptors from nine other insect species can be found at the silkworm database (http://silkworm.swu.edu.cn/silkdb.html).

**Figure 1:**
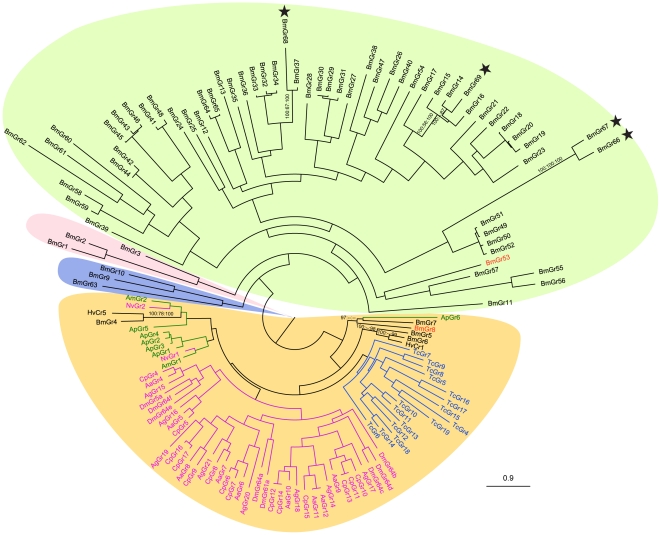
Molecular phylogeny comparing silkworm gustatory receptors with putative sugar receptors from nine insect species. 69 gustatory receptors from *B. mori* (BmGrn) and putative sugar receptors from *H. virescens* (HvCrn), *D. melanogaster* (DmGrn), *A. gambiae* (AgGrn), *A. aegypti* (AaGrn), *C. quinquefasciatus* (CpGrn), *A. mellifera* (AmGrn), *N. vitripennis* (NvGrn), *T. castaneum* (TcGrn) and *A. pisum* (ApGrn) were used to construct the phylogenetic tree. Bootstrap support for *B. mori* sugar receptors and four novel bitter receptors are shown as the percentage of 1 000 replications, if they are above 50%, of optimization criteria of Bayesian posterior probabilities, maximum likelihood and maximum parsimony. GRs from different insect orders are indicated by different colors. Stars indicate the novel four BmGrs. BmGr8 and BmGr53 are highlighted in red text. Clades are background shaded as follows: Orange, sugar receptor clade; Green, bitter receptor clade; Pink, CO_2_ receptor clade; Blue, DmGr43a homologues. See Materials and Methods for details of the phylogenetic analysis.

Phylogenetic analysis of all 69 BmGrs and putative sugar receptors from another nine insect species shows that Bombyx GRs BmGr4 to BmGr8 belong to the sugar receptor subfamily (Figure 1, Figure S1). The newly identified BmGrs are dispersed over three lineages in the putative bitter receptor subfamily (Figure 1), which in *Bombyx* has 59 BmGrs, an extraordinarily large single lineage. The silkworm bitter receptor lineage appears to have diverged and expanded later than the carbon dioxide subfamily, BmGr1-BmGr3, and DmGr43a orthologs BmGr9, BmGr10 and BmGr63. We selected BmGr8 as a representative of the putative sugar receptors for topological investigation. BmGr8 appears to represent a novel, phytophage-specific lineage within the sugar receptor family. The most closely related GRs, BmGr7 (partial GR, missing the N-terminus) and ApGr6 share respectively 25% and 18% amino acid sequence identity with BmGr8.

### BmGr8 is an inositol receptor

**Figure 2:**
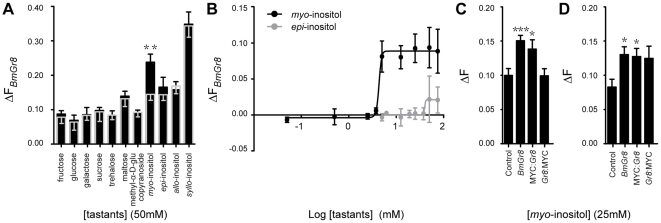
*In vitro* responsiveness of *BmGr8* to sugars and other tastants. *Sf9* cells were transfected with *BmGr8* in the PIB/V5-His vector. Cells were loaded with Fluo4 and the change in fluorescence (ΔF) was measured in response to tastants. ΔF was calculated as the ratio of change in fluorescence upon the addition of ligand relative to change in fluorescence following the addition of ionomycin. (A) Responses of *BmGr8* to eleven tastants at 50 mM. The mean responses of cells transfected with empty expression vector are indicated by the grey bar and grey error bars, mean responses of Gr8 transfected cells are indicated by the black bars and black error bars. (B) Log concentration-response curves for *BmGr8* responses to *myo*-inositol and *epi*-inositol. The average responses of cells transfected with empty expression vector have been subtracted. (C) Function of MYC-epitope tagged receptor responses to 50 mM *myo*-inositol. Control  =  empty vector, *Gr8*  =  BmGr8 transfected, MYC:*Gr8*  =  transfected with BmGr8 N-terminally fused to two MYC-epitope copies, *Gr8*:MYC  =  transfected with BmGr8 C-terminally fused to two MYC-epitope copies. (D) MYC-epitope tagging as for (C). Calcium imaging assay saline contains 170 mM D-glucose. See Table 2 for analysis of the statistical significance by two tailed Student's t test. Error bars in (A), (C) and (D) indicate the calculated error of the difference between means  =  SE (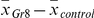), (B) indicate the standard error of the mean. *** p≤ 0.001, **p<0.01, *p<0.05.

In order to confirm that BmGr8 is a functional receptor we expressed *BmGr8* in *Sf9* cells and used quantitative imaging of calcium sensitive dye to characterize its ligand specificity. Three monosaccharides: glucose, fructose and galactose, three disaccharides: sucrose, maltose and trehalose, four inositol isomers and methyl-α-D-glucopyranoside, all at 50 mM, were used to characterize the ligand specificity of BmGr8. Of the eleven compounds tested, only 50 mM *myo*-inositol (p = 0.003) generated a response that deviated significantly from the control (Figure 2A, Table 1). A two-tailed Student's t-test indicated that the responses of BmGr8 to *myo*-inositol is higher than but not significantly different from (p = 0.072) the *epi*-inositol response and significantly higher than those of the other ten tastants (p<0.01) (Table 1). The response to *myo*-inositol had a threshold above 2 mM while the response to *epi-*inositol was only observed at or above 50 mM (Figure 2B). A lower limit for *myo*-inositol in fresh mulberry leaves of 1.6 mM was calculated from previous studies [52], [53]. The threshold of inositol responsiveness is therefore very close to the levels known to occur naturally in the silkworm's host plant.

**Table 1:** Statistical significance of differences in responses of BmGr8 to *myo*-inositol and each of ten other tastants at 50 mM using arcsine transformation and two-tailed Student's t-test.

A	*myo*-inositol	*epi-*inositol	*allo*-inositol	*syllo*-inositol	fructose	glucose	galactose	trehalose	maltose	sucrose	methyl-α-D-glucopyranoside
*VS* internal control											
t	3.06	0.45	0.30	0.09	0.52	0.01	0.12	0.17	0.30	0.15	0.17
df	62	54	77	68	76	69	84	65	61	74	56
p	0.003	0.768	0.766	0.925	0.607	0.989	0.903	0.866	0.768	0.877	0.864
B											
*VS myo-*inositol											
t		1.83	3.00	2.03	3.61	3.06	2.77	2.96	3.14	4.16	3.52
df		66	78	72	90	67	70	63	78	104	71
p		0.072	0.003	0.046	<0.001	0.003	0.007	0.004	0.002	<0.001	<0.001

(A) Statistical significance of the differences between each response and internal control (*Sf9* cells transfected with empty expression vector). (B) Statistical significances of the difference between the response to *myo*-inositol and each of the other tastants.

### Topology of insect gustatory receptors

**Figure 3:**
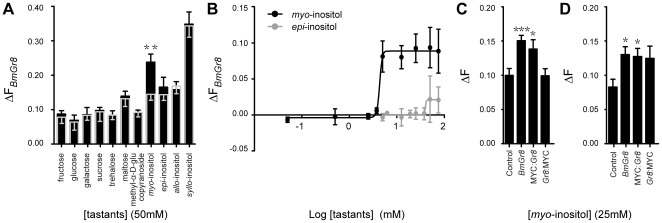
Summary histogram of the topological analysis of BmGrs. Transmembrane domain prediction of BmGrs using three algorithms, TMPred, HMMTOP and TMHMM. Histogram shows the number of genes predicted to have 3 to 9 transmembrane domains by each of the programs. Numbers in the graph show the percentage of predicted seven transmembrane proteins, which are predicted to have an intracellular N-terminus.

To investigate the membrane topology of lepidopteran and insect GRs generally, we first applied the algorithms TMPred, TMHMM and HMMTOP for predicting transmembrane domains (TMDs) to the known *Bombyx mori* GR sequences. For individual sequences, there was limited consensus among algorithms on the number and positions of TMDs. However, the most frequent prediction by each of the algorithms, across all GRs, is seven TMDs with an intracellular N-terminus (Figure 3, Table S1). For BmGr8, all three algorithms predicted an extracellular N-terminus, consistent with the typical topology of a GPCR, and up to nine TMDs with a consensus set of six, albeit with the first predicted TMD not well supported statistically. In order that our results have general validity for GR topology we selected a second gustatory receptor, BmGr53, encoded by only one exon in the genome [30], from the putative bitter receptor subfamily (Figure 1). BmGr53 was predicted by all three algorithms to have an intracellular N-terminus and seven TMDs (Table S1, Figure 5B).

Prior to performing immunocytochemical topological studies, we expressed MYC-tagged *BmGr8* in *Sf9* cells in order to determine whether this modified GR retained functionality. Using the same calcium-sensitive dye based protocol as previously, we found that cells transfected with native *BmGr8* and N-terminally MYC-tagged *BmGr8* (MYC:*Gr8*) showed statistically significant responses when compared to empty vector controls to 25 mM *myo*-inositol (Table 2 and Figure 2C). There was no statistically significant difference in responses between C-terminally tagged (*Gr8:*MYC) and the negative control. In experiments where the assay saline buffer contained 170 mM D-glucose, as previously used in other studies [13], [48], *Gr8:*MYC results were inconclusive as they showed a borderline but non-significant difference from the negative control and also a non significant difference from the *Gr8* responses to 50 mM inositol (Figure 2D, Table 2), suggesting the C-terminally tagged receptor is not completely inactive. However we cannot rule out the possibility that the C-term MYC tag renders the protein non functional due to the incorrect insertion of the receptor into the membrane. Similar experiments were not performed with *Gr53* because the appropriate ligand(s) is not known for this GR.

**Table 2:** Evaluation of the response differences between BmGr8 *VS* MYC tagged BmGr8 responding to 25 mM *myo*-inositol in low glucose (10 mM) assay saline, (A) and (B) and high glucose (170 mM) assay saline (C) and (D), using arcsine transformation and two-tailed Student's t-test.

	BmGr8	MYC:Gr8	Gr8:MYC
*In modified HBSS*			
(A) *VS* internal control			
t	3.87	2.29	0.05
df	152	102	91
p	<0.001	0.024	0.961
(B) *VS* BmGr8			
t		0.80	3.51
df		140	129
p		0.423	<0.001
*In 170 mM glucose assay saline*			
(C) *VS* internal control			
t	2.63	2.54	1.86
df	74	69	59
p	0.010	0.013	0.068
(D) *VS* BmGr8			
t		0.17	0.23
df		91	81
p		0.865	0.815

(A) and (C) Statistical significance of the differences between the responses against control. (B) and (D) Statistical significance of the differences between the responses of native BmGr8 and others.

**Figure 4:**
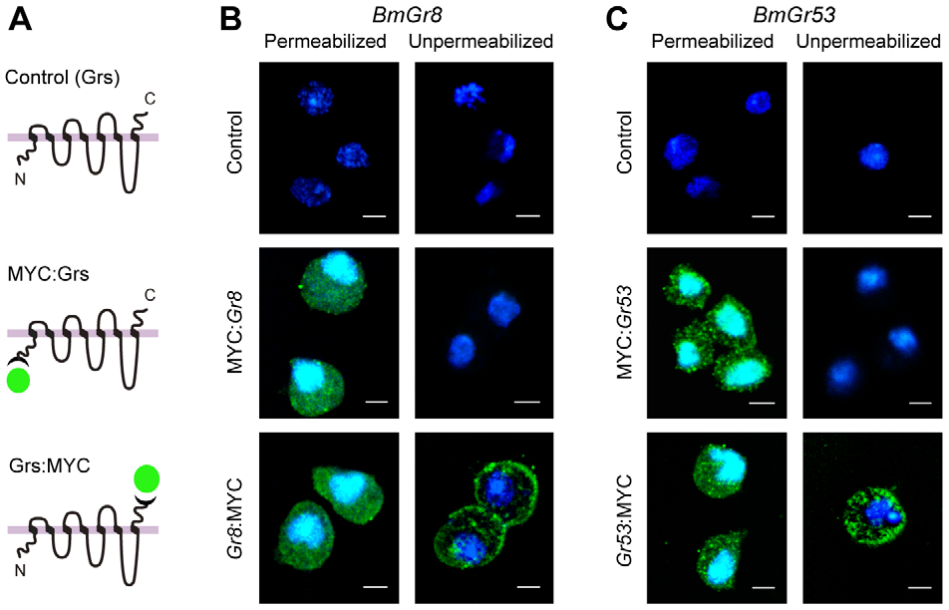
Immunochemical demonstration that the N-termini of BmGr8 and BmGr53 are cytoplasmic and the C-termini are extracellular. *BmGr8* was expressed in native form or fused with two MYC-epitopes at its N or C-termini. Cells were processed with mouse anti-MYC and Alexa-labelled anti-mouse antibodies to reveal the accessibility of the MYC antigen under permeabilised or unpermeabilised conditions. (A) Schematic of expression constructs for untagged GRs (controls), N-terminally MYC-tagged GRs (MYC:Grs) and C-terminally MYC-tagged GRs (Grs:MYC). (B) (C) Immunostaining of *BmGr8* and *BmGr53* in S2 cells under permeabilized and unpermeabilized conditions. Green indicates MYC-directed Alexa immunofluorescence. Blue indicates DAPI nuclear counter stain. Scale Bar  =  5 µm.

For topological studies, we expressed N- and C-terminally MYC-tagged fusions with BmGr8 and corresponding tagged fusions with BmGr53 in S2 cells since the strong auto-fluorescence observed in *Sf9* cells [13] tends to confound immunocytochemical experiments. Untagged receptors were used as a control (Figure 4). Strong green immunofluorescence was visualized from permeabilized cells when transfected with either MYC:*Gr8* or *Gr8:*MYC (Figure 4B). Similarly, strong green immunofluorescence was observed with permeabilised S2 cells expressing either MYC:*Gr53* or *Gr53:*MYC (Figure 4C). In contrast, when cells were not permeabilised, fluorescence was only seen in cells transfected with C terminally tagged GRs, *Gr8:*MYC or *Gr53:*MYC, and not from the N-terminally tagged constructs (Figure 4B and 4C), indicating that the N-termini of both proteins are located intracellularly and the C-termini of both proteins are extracellular.

**Figure 5:**
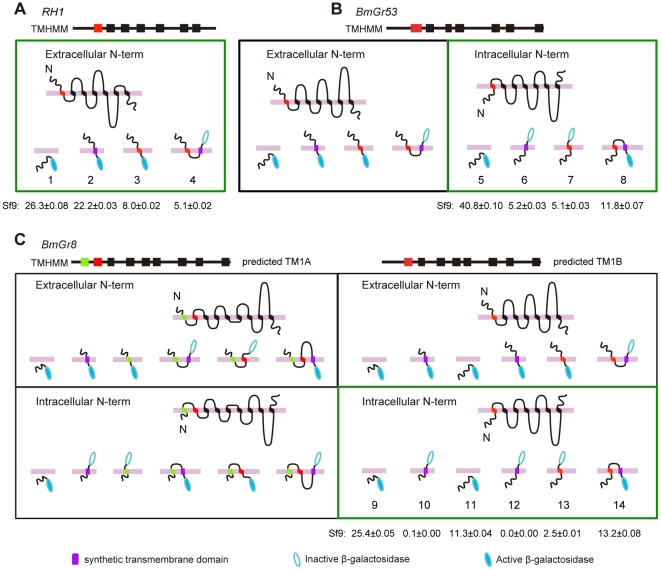
Use of β-galactosidase fusions to distinguish among possible GR topologies. Sequences encoding a series of N-terminal fragments of *Rhodopsin RH1*, *BmGr53* and *BmGr8* were fused at their 3′ ends through a linker to the gene encoding β-galactosidase and expressed in *Sf9* cells. Cells were fixed and the percentage of cells staining for β-galactosidase activity was scored, percentages ± standard deviation are indicated below construct numbers and in Table 3. In each case, selection of the N-terminal fragments used was based on known or putative locations of transmembrane domains (TMDs). (A) The topology of RH1 is well-established and correctly predicted by all three prediction algorithms, TMPred, TMHMM and HMMTOP (only TMHMM prediction is shown). RH1 was therefore used as a control. In this case, > 8% of cells transfected with fusions containing the N-terminus, N-terminus with a synthetic TMD, N-terminus with 1st predicted TMD or the N-terminus with 1st predicted TMD and an artificial TMD stained with X-gal. 5.1% of cells that were transfected with a fusion containing the N-terminus, the 1st predicted TMD and a synthetic TMD, which was taken as the threshold for inactive β-galactosidase. (B) The same procedure was used for BmGr53. Two possible results are shown, the expected staining pattern if N-terminal topology is the same as RH1, and the other where the topology is inverted. The latter staining pattern (outlined in green) was observed experimentally. (C) As for B, but with BmGr8. In this case, there is ambiguity over whether the first (TM1A) or second (TM1B) predicted TMD is the correct one. Therefore an additional set of constructs was made to cover both possibilities. Four possible experimental results are shown, encompassing expected staining patterns with intracellular or extracellular N-termini and the two possible first TMDs. Experimental results (outlined in green) conformed to a model with intracellular N-terminus and second predicted TMD which is the same pattern as for BmGr53. Purple rectangle, synthetic transmembrane domain; hollow blue ellipse, inactive β-galactosidase; solid blue ellipse, active β-galactosidase; N, N-terminus; red rectangle, the predicted first transmembrane domain with good support (i.e. TM1B for BmGr8); light green rectangle, the predicted first transmembrane (TM1A) of BmGr8 with poorer support. Cut off for scoring positive β-galactosidase was ≥8%, i.e. the lowest percentage observed for the positive control in A. The experiment was repeated in S2 cells with essentially identical results. Numbers identify the constructs for which quantitative data for both *Sf9* and S2 cells are shown in Table 3.

Given the novelty of the finding that the N-termini of BmGrs is intracellular, we used a β-galactosidase fusion technique [10] to confirm this result and to determine the authenticity of the putative transmembrane domains experimentally (Figure 5). We cloned a series of fragments of both receptors which were then fused at their 3′ ends, through a sequence encoding a 26 amino-acid linker to the LacZ gene, whose gene product β-galactosidase is only functional when expressed intracellularly [49], [50]. In addition, fusions were prepared that included a synthetic transmembrane domain. All constructs were transfected into *Sf9* (lepidopteran) and S2 (dipteran) cells, which were stained for β-galactosidase activity. The percentage of cells expressing active β-galactosidase was scored relative to positive and negative controls derived from fusions with the N-terminus of *Drosophila* rhodopsin RH1, a typical GPCR with a well-known topology (Figure 5A). In *Sf9* cells, β-galactosidase was scored as active if more than 8% of the cells stained blue, as this was the lowest percentage observed for the positive control *RH1* constructs (Table 3). In the case of BmGr53 (Figure 5B), the results observed were consistent with its having an intracellular N-terminus and confirmed the consensus first predicted TMD. An additional set of constructs was required for BmGr8 because of the ambiguity over which is the first TMD. The results (Figure 5C) were clearly consistent with BmGr8 also having its N-terminus in the cytoplasm. In this case, authenticity of the putative first predicted TMD was not supported by the results whereas the putative second TMD was shown to be the actual first TMD. Essentially identical results were observed in *Drosophila* S2 cells (Table 3) although the efficiency of expression was reduced with the lowest percentage of blue cells observed for a positive control being 4.7%. These results confirm the immunocytochemical results (Figure 4) indicating that both BmGr8 and BmGr53 have intracellular N-termini. Taken with the observation, based on our immunocytochemical experiments, that both GRs have an odd number of TMDs the results of the lac-Z fusions are consistent with both BmGr53 and BmGr8 having seven transmembrane domains, although they do not constitute unequivocal proof of this prediction.

**Table 3:** Percentages of *Sf9* or S2 cells staining for β-galactosidase activity for fusions with various fragments of RH1, BmGr53 and BmGr8.

Identities of fusion constructs	Sf9 cells		S2 cells	
	Mean percentage of blue cells (%)	SD	Mean percentage of blue cells (%)	SD
Met-LacZ	0.0	0.00	0.0	0.00
1	26.3	0.08	6.3	0.01
2	22.2	0.03	15.8	0.05
3	8.0	0.02	4.7	0.01
4	5.1	0.02	1.0	0.01
5	40.8	0.10	10.3	0.02
6	5.2	0.03	1.7	0.00
7	5.1	0.03	0.2	0.00
8	11.8	0.07	5.9	0.01
9	25.4	0.05	20.1	0.02
10	0.1	0.00	0.0	0.00
11	11.3	0.04	12.0	0.00
12	0.0	0.00	0.1	0.00
13	2.5	0.01	0.7	0.00
14	13.2	0.08	4.8	0.00

Met-LacZ: a blank construct containing *LacZ* without the start codon in PIB/V5-His. Numbers 1–14 represent the constructs depicted in Figure 5. 1–4 from RH1. 5–8 from BmGr53. 9–14 from BmGr8. These data are the basis of the summary shown in Figure 5 and should be read in conjunction with that figure. Mean and standard deviation of 12 wells (3 biological repeats of 4 replicates each).

## Discussion

Insect chemoreceptors were initially identified by their predicted structural similarity to the seven transmembrane chemosensory GPCRs of vertebrates [54], [55]. Mammalian olfactory receptors have a typical GPCR topology with an extracellular N-terminus and intracellular C-terminus [56], [57], [58], [59], [60], [61], [62]. More recent computational analysis predicted that the membrane topology of insect odorant receptors is different from that of members of the GPCR superfamily [39] and they have been shown to have an inverted topology, relative to GPCRs, with their N-termini intracellular [10], [11], [13], [63]. Insect ORs are thought to function as odour-gated ion channels [12], [14] although there is likely to be a modulatory role for G-proteins and second messengers downstream of the ORs [64], [65]. Here we have shown that two lepidopteran GRs, BmGr8 and BmGr53, share the same inverted topology as insect ORs, indicating that insect GRs are not classical GPCRs and may function in a similar way to insect ORs. G-proteins have been shown to play a role in the perception of some sweet tastants [16] and for CO_2_ reception in *Drosophila* [19], however they were not absolutely required for electrophysiological and behavioral responses. These observations together with the topological data from this study support the hypothesis that the dual activation model [65] proposed for insect ORs may also extend to the GR family.

This study is also the first report linking specific ligands to gustatory receptor function in a member of the Lepidoptera or indeed in any non-dipteran insect. We demonstrate that the gustatory receptor, BmGr8, responds to *myo*-inositol and, to a lesser extent, *epi*-inositol *in vitro*. Direct quantitative comparison of the magnitude of the response in this system with olfactory receptors is confounded by the variable level of expression of each receptor and wide variation in efficacies of different receptor-ligand pairs. However the ΔF we measured for BmGR8 is within the range previously reported for ORs (0.1–0.4) [13], [48]. Interestingly, these responses only required the expression of *BmGr8*. *Drosophila* sugar receptors, such as Gr5a, are thought to act as multimers *in vivo* as they require the broadly expressed Gr64f co-receptor for proper detection of sugars [23]. Nevertheless, expression of only *Gr5a* in cultured S2 cells was sufficient to mediate responses to trehalose [20], suggesting either that homomeric receptors retain function or that these insect cell lines express an endogenous co-receptor, as seen with the olfactory co-receptor, *Orco* (recently renamed from *DmOr83b*) [66], which is expressed endogenously in *Sf9* cells [13]. *B. mori* do not have an orthologue of the *Drosophila* Gr64f co-receptor, however we cannot rule out that a different co-receptor is expressed in *Sf9* cells, or that other receptors which may be co-expressed *in vivo* could alter the specificity of BmGr8.

BmGr8 is predicted to fall within the sugar-responsive clade of GRs but it did not respond to a number of conventional reducing sugars. Inositol (cyclohexane-1,2,3,4,5,6-hexol) is somewhat similar to sugar alcohols but generates little or no sweetness perception in humans. *Myo*-inositol is found ubiquitously in plants [67] and plays an important role in animal metabolism, including as a precursor for synthesis of inositol lipids and thence the second messenger inositol triphosphate [68], [69], [70], [71], [72], [73]. *Myo*-inositol cannot be synthesized by all insects and is an essential nutrient in some lepidopteran species, including *B. mori* [3], [74], [75]. *Myo*-inositol has long been known to stimulate different aspects of feeding in Lepidoptera such as prolonged feeding in *B.mori* larvae [76], [77], [78], [79], initiation of feeding in *Manduca sexta* [3], [80], [81] and induction of the proboscis extension reflex in *Helicoverpa armigera* [82]. In *B. mori,* one neuron in the lateral styloconic sensillum of the maxillary galea responds to *myo*-inositol and *epi*-inositol whereas another neuron in the same sensillum responds to *allo*-inositol [72]. In our study, which may not accurately reflect *in vivo* sensitivity, BmGr8 responded to over 2 mM *myo*-inositol and 50 mM *epi*-inositol. These indicate BmGr8 would be a candidate to be one of the receptors expressed in the lateral styloconic sensilla. The orthologs, BmGr7 and ApGr6, in the same lineage would be obvious candidates as inositol receptors, possibly in the case of BmGr7 for other isomers of inositol.

## Supporting Information

Figure S1 **Multiple alignment of insect sugar receptors by Clustal X.**
(PDF)

Figure S2 **Expression constructs design and primers used in this study.**
(DOC)

Table S1 **Transmembrane domain prediction of BmGrs using TMPred, HMMTOP and TMHMM.**
(DOC)
