# Correction: Topological and Functional Characterization of an Insect Gustatory Receptor

**DOI:** 10.1371/annotation/df99f702-e321-4eb6-911a-57fe12443e6f

**Published:** 2011-09-23

**Authors:** Hui-Jie Zhang, Alisha R. Anderson, Stephen C. Trowell, A-Rong Luo, Zhong-Huai Xiang, Qing-You Xia

The complete funding information is: "This work was supported by a grant from the the National Natural Science Foundation of China (30972147), the Ministry of Education of the People's Republic of China Program for Changjiang Scholars and Innovative Research Team in University (No. IRT0750), CSIRO and the Doctoral Innovation Fund of Southwest University (kb2008002). The funders had no role in study design, data collection and analysis, decision to publish, or preparation of the manuscript." 

