# Supplementary material for: Topological and Functional Characterization of an Insect Gustatory Receptor
Source: PLoS One. 2011 Aug 29;6(8):e24111. doi: 10.1371/journal.pone.0024111 (PMC3163651; doi:10.1371/journal.pone.0024111)

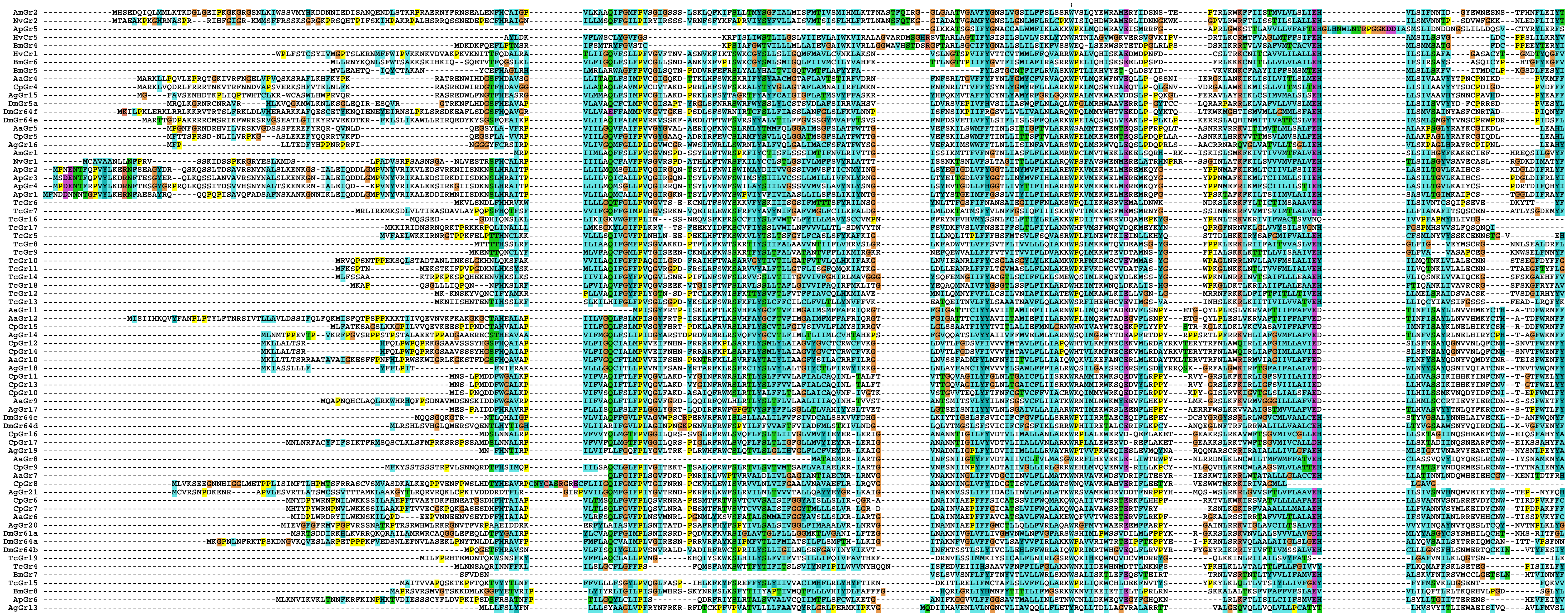
AmGr2
NvGr2
ApGr5
HvCr5
BmGr4
HvCr1
BmGr6
BmGr5
AaGr4
CpGr4
AgGr15
DmGr5a
DmGr64f
DmGr64e
AaGr5
CpGr5
AgGr16
AmGr1
NvGr1
ApGr2
ApGr3
ApGr4
ApGr1
TcGr6
TcGr7
TcGr16
TcGr17
TcGr5
TcGr8
TcGr9
TcGr10
TcGr11
TcGr14
TcGr18
TcGr12
TcGr13
AaGr11
AaGr12
CpGr15
AgGr14
CpGr12
CpGr14
AaGr10
AgGr18
CpGr11
CpGr13
CpGr10
AaGr9
AgGr17
DmGr64c
DmGr64d
CpGr16
CpGr17
AgGr19
AaGr8
CpGr9
AaGr7
CpGr8
AgGr21
CpGr6
CpGr7
AaGr6
AgGr20
DmGr61a
DmGr64a
DmGr64b
TcGr19
TcGr4
BmGr7
TcGr15
BmGr8
ApGr6
AgGr13

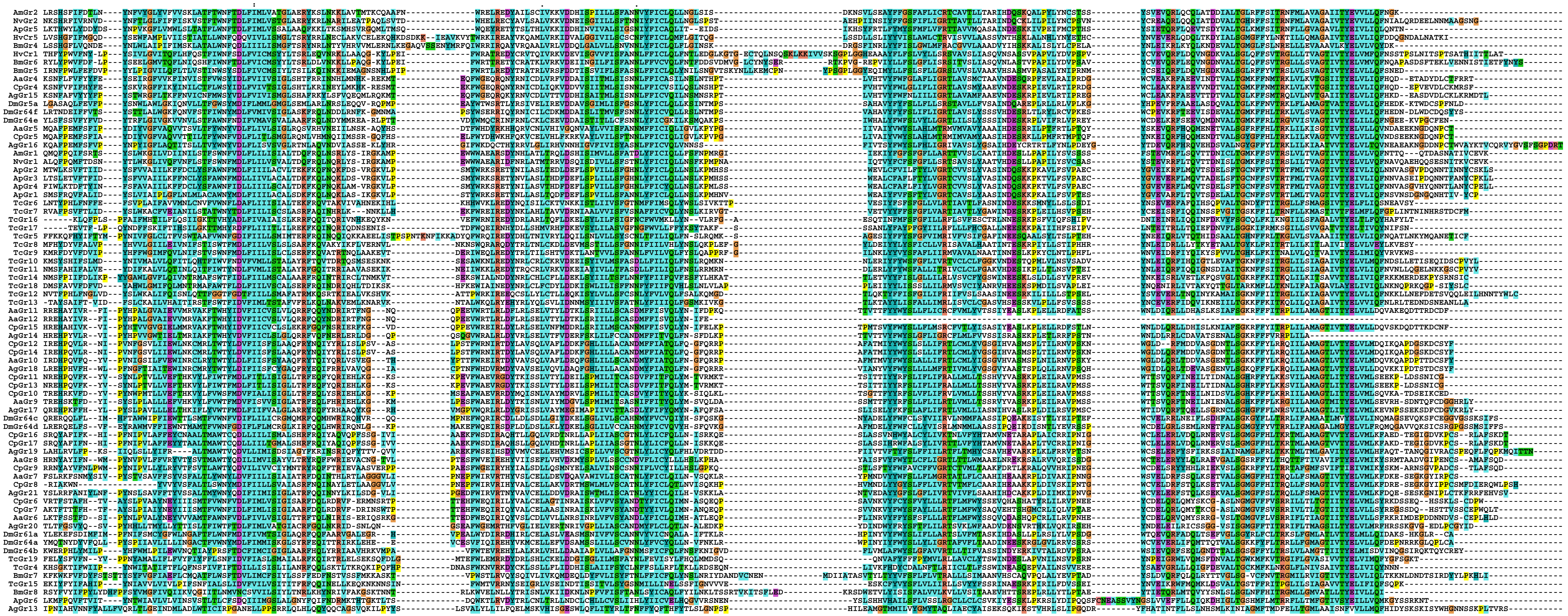

Supplement: Figure S1 — Multiple alignment of insect sugar receptors by Clustal X. (PDF) [file pone.0024111.s001.pdf]
