# Supplementary material for: Topological and Functional Characterization of an Insect Gustatory Receptor
Source: PLoS One. 2011 Aug 29;6(8):e24111. doi: 10.1371/journal.pone.0024111 (PMC3163651; doi:10.1371/journal.pone.0024111)
Supplement: Figure S2 — Expression constructs design and primers used in this study. (DOC) [file pone.0024111.s002.doc]

**Genes clone from cDNA:**

*>BmGr8*

Fwd- GGGGTACCATGGCTCCTCGATCAGTTCGATC,

Rev- TCCCCGCGGGGA TTAAATTTGAAGTAATACTATTTCG

*>BmGr53*

Fwd- GGGGTACCATGATGGCTCACATAAA

Rev- TCCCCGCGGGGA TTAGACAAAATGAGAGAGTTGAATAATCA

**For β-galactosidase fusion experiments:**

Kpn I

N tail

BamH I

Xho I

Sac II

Synthetic TM

TrpS linker

Lac Z

>synthetic transmembrane domain

GGATCCGAAAGCTGGCAAAGGGCTCTTGTCCTGCTAATCGTACTACTCTTCATCGTCATCTTCGTTATTACTGTTTTGTTCGTCATACTCGAGGG

>LacZ

Fwd-CCCTCGAGGTGGGTGAAGACCAGAAACAGCACCTCGAACTGAGCCGCGATATTGCCCAGCGTTTCAACGCGCTGTATGGCGAGATCGATCCCGTCGTTTTACAACGTCG

Rev- TCCCCGCGGGGTTATTATTATTTTTGACACCAGACCAACTGG

**For MYC-epitope tagged genes’ constructs:**

**2X MYC in the downstream of *BmGr8***

Kpn I

Sac II

2X MYC

*BmGr8*

>BmGr8

Fwd- GGGGTACCATGGCTCCTCGATCAGTTCGATC

Rev- TCCCCGCGGGGATTAAAGGTCTTCTTCGGAGATAAGCTTTTGTTCAAGGTCTTCTTCGGAGATAAGCTTTTGTTCAATTTGAAGTAATACTATTTCGTACGT

**2X MYC in the upstream of *BmGr8***

Kpn I

Sac II

2X MYC

*BmGr8*

>BmGr8

Fwd- GGGGTACCATGGAACAAAAGCTTATCTCCGAAGAAGACCTTGAACAAAAGCTTATCTCCGAAGAAGACCTTCTGGCTCCTCGATCAGTTCGATCAATGGTCG

Rev- TCCCCGCGGGGATTAAATTTGAAGTAATACTATTTCG

**2X MYC in the downstream of *BmGr53***

Xba I

BamH I

2X MYC

*BmGr53*

>BmGr53

Fwd- CGGGATCC ATGGCTCACATAAAAGATGAAAATC

Rev- ACGTCTAGATTAAAGGTCTTCTTCGGAGATAAGCTTTTGTTCAAGGTCTTCTTCGGAGATAAGCTTTTGTTC

GACAAAATGAGAGAGTTGAATAATCA

**2X MYC in the upstream of *BmGr53***

Xba I

BamH I

2X MYC

*BmGr53*

>BmGr53

Fwd- CGGGATCCATGGAACAAAAGCTTATCTCCGAAGAAGACCTTGAACAAAAGCTTATCTCCGAAGAAGACCTT CTGGCTCACATAAAAGATGAAAATC

Rev- ACGTCTAGATTAGACAAAATGAGAGAGTTGAATAATCA

**For EGFP tagged genes’ constructs:**

Kpn I

Sac II

EGFP

*BmGr8*

EcoR I

>BmGr8

Fwd- GGGGTACCCCATGGCTCCTCGATCAGTTCGATC

Rev- GAATTCAATTTGAAGTAATACTATTTCG

>EGFP

Fwd- GAATTCGTGAGCAAGGGCGAGGAG

Rev- TCCCCGCGGGGATTACTTGTACAGCTCGTCCATG

Kpn I

Sac II

EGFP

*BmGr8*

EcoR I

>EGFP

Fwd- GGGGTACCCCATGGTGAGCAAGGGCGAGGAGCTG

Rev- GAATTCCTTGTACAGCTCGTCCATGCCG

>BmGr8

Fwd- GAATTCGCTCCTCGATCAGTTCGATC

Rev- TCCCCGCGGGGATTAAATTTGAAGTAATACTATTTCG
