# Supplementary material for: Topological and Functional Characterization of an Insect Gustatory Receptor
Source: PLoS One. 2011 Aug 29;6(8):e24111. doi: 10.1371/journal.pone.0024111 (PMC3163651; doi:10.1371/journal.pone.0024111)
Supplement: Table S1 — Transmembrane domain prediction of BmGrs using TMPred, HMMTOP and TMHMM. (DOC) [file pone.0024111.s003.doc]

**Table S1** Transmembrane domain prediction of BmGrs using TMPred, HMMTOP and TMHMM

|  | TMPred | | | HMMTOP | | | TMHMM | | |
| --- | --- | --- | --- | --- | --- | --- | --- | --- | --- |
| Genes | TMs | N-in | N-out | TMs | N-in | N-out | TMs | N-in | N-out |
| RH1 | 7 |  | √ | 7 |  | √ | 7 |  | √ |
| BmGr1 |  |  |  |  |  |  |  |  |  |
| BmGr2 |  |  |  |  |  |  |  |  |  |
| BmGr3 | 7 | √ |  | 8 |  | √ | 6 | √ |  |
| BmGr4 | 6 | √ |  | 7 | √ |  | 6 | √ |  |
| BmGr5 | 6 |  | √ | 6 |  | √ | 6 |  | √ |
| BmGr6 | 8 |  | √ | 8 |  | √ | 6 | √ |  |
| BmGr7 |  |  |  |  |  |  |  |  |  |
| BmGr8 | 8 |  | √ | 9 |  | √ | 9 |  | √ |
| BmGr9 | 8 | √ |  | 7 | √ |  | 7 |  | √ |
| BmGr10 | 8 |  | √ | 7 | √ |  | 6 | √ |  |
| BmGr11 | 7 | √ |  | 7 | √ |  | 7 | √ |  |
| BmGr12 | 7 | √ |  | 6 |  | √ | 7 | √ |  |
| BmGr13 | 7 | √ |  | 7 | √ |  | 5 | √ |  |
| BmGr14 | 8 |  | √ | 8 |  | √ | 8 | √ |  |
| BmGr15 | 8 | √ |  | 8 |  | √ | 8 | √ |  |
| BmGr16 | 7 |  | √ | 8 |  | √ | 7 | √ |  |
| BmGr17 | 9 | √ |  | 9 | √ |  | 9 | √ |  |
| BmGr18 | 7 |  | √ | 8 |  | √ | 7 | √ |  |
| BmGr19 | 7 | √ |  | 7 | √ |  | 7 | √ |  |
| BmGr20 | 8 |  | √ | 7 | √ |  | 6 |  | √ |
| BmGr21 | 7 |  | √ | 8 |  | √ | 7 | √ |  |
| BmGr22 | 7 |  | √ | 7 | √ |  | 7 | √ |  |
| BmGr23 | 6 | √ |  | 6 |  | √ | 5 | √ |  |
| BmGr24 | 5 |  | √ | 6 | √ |  | 7 | √ |  |
| BmGr25 | 8 |  | √ | 8 |  | √ | 8 |  | √ |
| BmGr26 | 7 | √ |  | 7 | √ |  | 7 | √ |  |
| BmGr27 | 6 |  | √ | 7 | √ |  | 3 | √ |  |
| BmGr28 | 7 | √ |  | 7 | √ |  | 5 | √ |  |
| BmGr29 | 7 |  | √ | 7 | √ |  | 7 | √ |  |
| BmGr30 | 7 |  | √ | 7 | √ |  | 7 | √ |  |
| BmGr31 |  |  |  |  |  |  |  |  |  |
| BmGr32 | 8 |  | √ | 8 |  | √ | 8 |  | √ |
| BmGr33 | 7 |  | √ | 8 |  | √ | 8 |  | √ |
| BmGr34 | 7 | √ |  | 8 |  | √ | 6 | √ |  |
| BmGr35 | 8 |  | √ | 8 |  | √ | 6 | √ |  |
| BmGr36 | 8 | √ |  | 8 |  | √ | 8 |  | √ |
| BmGr37 |  |  |  |  |  |  |  |  |  |
| BmGr38 | 7 |  | √ | 8 |  | √ | 7 | √ |  |
| BmGr39 |  |  |  |  |  |  |  |  |  |
| BmGr40 | 7 | √ |  | 8 |  | √ | 6 | √ |  |
| BmGr41 | 7 | √ |  | 8 | √ |  | 7 | √ |  |
| BmGr42 | 8 |  | √ | 8 |  | √ | 7 | √ |  |
| BmGr43 |  |  |  |  |  |  |  |  |  |
| BmGr44 |  |  |  |  |  |  |  |  |  |
| BmGr45 | 7 | √ |  | 7 | √ |  | 7 | √ |  |
| BmGr46 | 7 | √ |  | 7 | √ |  | 7 | √ |  |
| BmGr47 | 7 | √ |  | 7 | √ |  | 7 | √ |  |
| BmGr48 | 7 | √ |  | 8 | √ |  | 6 |  | √ |
| BmGr49 |  |  |  |  |  |  |  |  |  |
| BmGr50 | 6 | √ |  | 9 |  | √ | 5 | √ |  |
| BmGr51 | 6 | √ |  | 8 | √ |  | 7 | √ |  |
| BmGr52 | 7 |  | √ | 8 | √ |  | 7 | √ |  |
| BmGr53 | 5 | √ |  | 7 | √ |  | 7 | √ |  |
| BmGr54 | 6 |  | √ | 8 |  | √ | 5 | √ |  |
| BmGr55 | 8 | √ |  | 7 | √ |  | 7 | √ |  |
| BmGr56 | 7 | √ |  | 7 | √ |  | 6 |  | √ |
| BmGr57 | 6 |  | √ | 7 | √ |  | 6 | √ |  |
| BmGr58 | 7 | √ |  | 7 | √ |  | 6 | √ |  |
| BmGr59 | 7 |  | √ | 7 | √ |  | 6 | √ |  |
| BmGr60 | 7 |  | √ | 7 | √ |  | 5 |  | √ |
| BmGr61 | 7 |  | √ | 7 | √ |  | 4 | √ |  |
| BmGr62 | 7 |  | √ | 9 |  | √ | 7 | √ |  |
| BmGr63 | 5 |  | √ | 7 | √ |  | 6 |  | √ |
| BmGr64 | 7 | √ |  | 7 | √ |  | 7 | √ |  |
| BmGr65 |  |  |  |  |  |  |  |  |  |
| BmGr66 |  |  |  |  |  |  |  |  |  |
| BmGr67 |  |  |  |  |  |  |  |  |  |
| BmGr68 |  |  |  |  |  |  |  |  |  |
| BmGr69 |  |  |  |  |  |  |  |  |  |
